# Change point models for cognitive tests using semi-parametric maximum likelihood

**DOI:** 10.1016/j.csda.2012.07.024

**Published:** 2013-01

**Authors:** Ardo van den Hout, Graciela Muniz-Terrera, Fiona E. Matthews

**Affiliations:** a Department of Statistical Science, University College London, 1–19 Torrington Place, London WC1E 7HB, UK; b MRC Unit for Lifelong Health and Ageing, London, UK; c MRC Biostatistics Unit, Institute of Public Health, Cambridge, UK

**Keywords:** Beta-binomial distribution, Latent class model, Mini-mental state examination, Random-effects model

## Abstract

Random-effects change point models are formulated for longitudinal data obtained from cognitive tests. The conditional distribution of the response variable in a change point model is often assumed to be normal even if the response variable is discrete and shows ceiling effects. For the sum score of a cognitive test, the binomial and the beta-binomial distributions are presented as alternatives to the normal distribution. Smooth shapes for the change point models are imposed. Estimation is by marginal maximum likelihood where a parametric population distribution for the random change point is combined with a non-parametric mixing distribution for other random effects. An extension to latent class modelling is possible in case some individuals do not experience a change in cognitive ability. The approach is illustrated using data from a longitudinal study of Swedish octogenarians and nonagenarians that began in 1991. Change point models are applied to investigate cognitive change in the years before death.

## 1 Introduction

The scale of a cognitive test is often discrete. A typical example is the Mini-Mental State Examination (MMSE Folstein et al., 1975) which has integer scoring. The MMSE is a questionnaire for screening dementia and has items on, for instance, language and memory. Scores for each of the questions are added up to obtain a final integer sum score ranging from 0 to 30.

This paper discusses and extends methodology for random-effects change point models for longitudinal data on cognitive tests. A change point model assumes a stochastic process over time that shows a one-off change in direction, see, e.g., Dominicus et al. (2008). Change points are sometimes called *turning points* (McArdle and Wang, 2008) or *break points* (Stasinopoulos and Rigby, 1992; Muggeo, 2008). Models with more than one change point are typically applied to time series data, see, e.g., Bauwens and Rombouts (2012).

Cognitive test data are often analysed using the normal distribution, see, e.g., Laukka et al. (2006). This may be problematic for many reasons. We illustrate this with the MMSE. If the normal distribution is used, then prediction of MMSE scores is not restricted to the original test scale and this can lead to interpretation problems when predicted scores are outside the scale 0–30. Ceiling effects further undermine the use of the normal distribution as these effects cause a dependency between residuals and fitted values which violate model assumptions. In the MMSE, a majority of observed sum scores in the range 28–30 is indicative of a ceiling effect.

The wider framework of our statistical modelling is that of random-effects growth models with a non-linear link between the response and the predictor, where the predictor is non-linear in the parameters. We propose change point regression models with discrete probability distributions—appreciating the essential discrete nature of cognitive test data. Dependencies within the repeated measurements of an individual are dealt with by using random effects. In addition, we formulate a latent class model, which allows a priori for two latent groups in the data: one group where the cognitive process changes over time, and one group where the process is stable. For the first group a change point model is formulated. For both groups random-effects are included in the predictors. Special attention is given to residual diagnostics for model validation.

A common choice for the distribution of the random effects in regression models for longitudinal data is the multivariate normal (Rabe-Hesketh and Skrondal, 2009). As an alternative, the models in this paper assume a non-parametric distribution for the regression coefficients combined with a parametric distribution for the change point. Non-parametric maximum likelihood estimation of random effects in models with linear predictors has been discussed in Aitkin (1999), Molenberghs and Verbeke (2005), and Muthén and Asparouhov (2009). By adopting the non-parametric approach, the assumption of normality for the random effects is avoided, and optimizing the likelihood is computationally less demanding. The specification of the distribution of the random effects does not always have an impact on the estimation of the parameters of interest (Aitkin, 1999), but there are examples where the normality assumption leads to bias (Muthén and Asparouhov, 2009). The main advantage of the non-parametric approach is that it works well when the effects are normally distributed *and* when they are not. We extend the non-parametric approach to models with non-linear predictors. The choice of the parametric distribution for the change point is a truncated normal, which is specific to our application.

A general way to define a class of change point models is to assume a polynomial regression model of degree $d_{1}$ before the change point, and a polynomial regression model of degree $d_{2}$ after, see, e.g., Rudoy et al. (2010). The *broken-stick model* is a member of this class: there are two linear parts, one before and one after the change point, and continuity is imposed such that the linear parts intersect at the change point. The broken-stick model can also be described as a piecewise linear model with one free knot. It has been used in many applications, e.g. in AIDS research (Kiuchi et al., 1995), in social statistics (Cohen, 2008), and in medical statistics, (Hall et al., 2003; Muniz-Terrera et al., 2011).

Van den Hout et al. (2011) introduced a model where the two linear parts are bridged by a third-degree polynomial which induces a smooth transition between the parts. Similarities between this model and *bent-cable* regression as presented in Chiu et al. (2006) will be investigated. The class of models introduced by Bacon and Watts (1971) will also be considered. The current paper can be seen as a follow-up to Van den Hout et al. (2011) in the sense that we improved upon the choice of the change point predictor and its selection, and improve the modelling with respect to the distributional assumptions for the conditional response and the random effects.

In the application, change point models will be used to investigate features of cognitive change in the older population in the years before death. The modelling is tailored to the terminal decline hypothesis which states that individuals experience a change in the rate of decline of cognitive function before death (Riegel and Riegel, 1972). Where there is a decline, we are interested in the timing of the rate change, and in its shape. Longitudinal MMSE data are available from the Swedish OCTO-Twin study (McClearn et al., 1997). In this longitudinal study of aging (1991–2009), MMSE scores are recorded over time. Because almost all death times are available (94%) in this study, we assume that the effect of ignoring the data of the survivors is negligible and we analyse the data of those who died using years-to-death as the time scale.

Section 2 introduces the various change point models and choices for the conditional distribution. In Section 3, semi-parametric likelihood inference is discussed. Section 4 extends methodology to a latent class model that distinguishes a stable class versus a change class for cognitive function over time. In Section 5, data from the OCTO study are analysed. Section 6 concludes the paper.

## 2 Models

Given response variable Y, predictor η, link function l(), and time t as explanatory variable, the conditional mean of Y is given by E[Y|t]=l(η) with η=h(t,β,τ), where h() is the function that defines the predictor using coefficient vector $\beta =\left(\beta_{0},\beta_{1},\beta_{2}\right)$ and change point τ.

The predictors in this section are non-linear in the change point parameter τ. Although the same notation for the regression coefficients is used for the various change point predictors, the interpretation of the coefficients varies across the models.

Extensions can be defined in a straightforward manner by including additional explanatory variables x to capture observed heterogeneity. In that case, η=h(t,x,β,τ).

The structure of the models in this section is similar to that of generalised non-linear random-effects models. The difference is that using the beta-binomial distribution for the response defines a model outside the natural exponential family, see Agresti (2002).

### 2.1 Predictors

The broken-stick model is given by

(1) $$\eta_{BS}=h_{BS}\left(t,\beta ,\tau \right)=\left\{ \begin{array}{cc} \beta_{0}+\beta_{1}t & t<\tau \\ \beta_{0}+\beta_{1}\tau +\beta_{2}\left(t-\tau \right) & t\geq \tau \text{.} \end{array}\right.$$

In this model the change is not smooth. As a function of t, there is no derivative of $h_{BS}$ at t equal to τ.

Bacon and Watts (1971) introduced a class of smooth change point models where the mean of the response is described by a non-linear predictor. The same idea can be used when link functions are applied. We define

(2) $$\eta_{BW}=h_{BW}\left(t,\beta ,\tau \right)=\beta_{0}+\beta_{1}\left(t-\tau \right)+\beta_{2}\left(t-\tau \right)\tanh\left(\left(t-\tau \right)/\gamma \right)\text{,}$$

for transition parameter γ>0. In this model, the hyperbolic tangent (tanh) is a transition function. For γ close to zero, the model implies a quick transition, whereas for large values, the change is very gradual. The effect of γ depends on the link function and the scale of the variable. A reasonable value of γ for the identity link will not necessarily be the best one for the logit link. The Bacon–Watts model (2) implies a smooth change for the first and second derivative with respect to t.

A possible alternative to model (2) is the polynomial model introduced in Van den Hout et al. (2011) where a third-degree polynomial is fitted between two linear parts. Transition parameter ϵ>0 specifies the interval between the two linear parts that is bridged by the curve. The model is given by

(3) $$\eta_{PL}=h_{PL}\left(t,\beta ,\tau \right)=\left\{ \begin{array}{cc} \beta_{0}+\beta_{1}t & t<\tau \\ g\left(t|\beta ,\tau ,\epsilon \right) & \tau \leq t<\tau +\epsilon \\ \beta_{2}+\beta_{3}t & \tau +\epsilon \leq t\text{,} \end{array}\right.$$

where g is a third degree polynomial. Smoothness of the transition is implied by imposing the following constraints for g:

$$g\left(\tau \right)=\beta_{0}+\beta_{1}\tau \;g\left(\tau +\epsilon \right)=\beta_{2}+\beta_{3}\left(\tau +\epsilon \right)$$

$$\left(\frac{\partial }{\partial t}g\right)\left(\tau \right)=\beta_{1}\;\left(\frac{\partial }{\partial t}g\right)\left(\tau +\epsilon \right)=\beta_{3}\text{.}$$

The top two constraints imply continuity between the polynomial curve and the two linear parts, and the bottom two constraints imply smoothness at the points where the polynomial curve connect to the two linear parts. The set of constraints uniquely defines g which is obtainable by solving a system of four linear equations with four unknown parameters. Polynomial g has a first derivative with respect to changing t, but the second derivative is not defined for all t. Note that the scale of the transition parameter ϵ is the scale of t and its interpretation is not affected by the choice of the link function.

It is possible to add a constraint to (3) such that the two linear parts intersect at the midpoint of the bridge between the two parts, i.e., at τ+ϵ/2. This constraint implies $\beta_{2}=\beta_{0}+\beta_{1}\left(\tau +\epsilon /2\right)-\beta_{3}\left(\tau +\epsilon /2\right)$, and the model becomes effectively a smoothed broken-stick model. A possible next step is to replace $\beta_{2}$ by $\beta_{2}-\nu$, where extra parameter ν<0 is called the *offset* and quantifies a drop between the first linear part and the second linear part, see also Section 2.2. Because the definition of g still applies, g will also in this case smoothly bridge the interval ϵ between the two linear parts. This parameterization is of interest for a cognitive process that shows a drop in cognitive function followed by a stable trend after the drop.

Bent-cable regression is a change point model introduced by Tishler and Zang (1981) and further developed and investigated by Chiu et al. (2006). The model can be seen as a smoothed broken-stick model and is given for δ>0 by

(4) $$\eta_{BC}=h_{BC}\left(t,\beta ,\tau \right)=\left\{ \begin{array}{cc} \beta_{0}+\beta_{1}t & t<\tau -\delta \\ \beta_{0}+\beta_{1}t+\beta_{2}\frac{{\left(t-\tau +\delta \right)}^{2}}{4\delta } & \tau -\delta \leq t\leq \tau +\delta \\ \beta_{0}+\left(\beta_{1}+\beta_{2}\right)t-\beta_{2}\tau & \tau +\delta <t\text{.} \end{array}\right.$$

The basic idea in bent-cable regression is that the kink in the broken-stick model is replaced by a quadratic bend with midpoint τ and half-width δ. If the aim is a smoothed broken-stick model, then (4) is to be preferred over (3) since the latter requires fitting a three-degree polynomial where a quadratic curve suffices. If model (3) includes the restriction on $\beta_{2}$ such that the two linear parts in (3) intersect at the midpoint of the bridge between the parts, then (3) will yield the same fit as (4).

All four change point models can be readily extended to random-effects models. For longitudinal data, regression parameters can be defined for individual i by $\beta_{i}$, and $\tau_{i}$, and a population distribution for these individual-specific parameters can be imposed.

Smooth change point models were introduced in the literature because fixed-effects piecewise linear models (such as the broken-stick model above) have no continuous first-order partial derivatives for the change points and this hampers the use of gradient techniques in the estimation of the parameters (Tishler and Zang, 1981). When a change point is modeled as a random effect, this problem disappears since the parameters of the change point distribution are estimated instead of a fixed-effect change point. The reason why smooth models are of interest in the context of cognitive tests is that the imposed shape of the cognitive change is in most cases more realistic than the sudden kink that is implied by the broken-stick model.

### 2.2 Graphical illustration of models

**Fig. 1:**
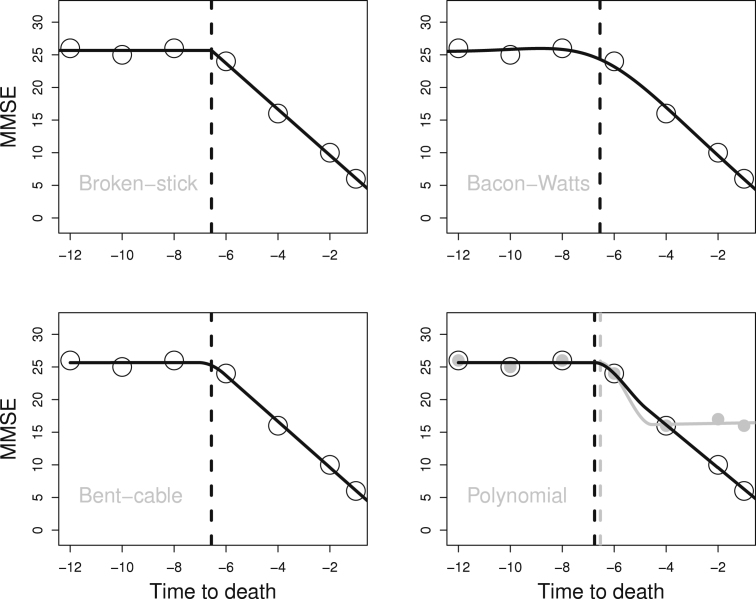
Toy data representing longitudinal MMSE scores for one individual. Vertical lines for the location of the estimated change point τ. Grey data points for an MMSE trend which stabilises after change (offset ν estimated at −9.56).

We illustrate the change point models using toy data for one individual. The data in Fig. 1 show a terminal decline in the years before death on the MMSE scale. Fig. 1 also depicts the fit of fixed-effects models. Note that the time scale is years to death, in the sense that −8 on the horizontal axis, for example, means 8 years before death. The Bacon–Watts, the polynomial, and the bent-cable are specified conditional on fixed transition parameters γ=3,ϵ=2, and δ=1/2. We use the normal distribution for the conditional distribution of the MMSE and use maximum likelihood estimation.

The shape of the Bacon–Watts model is highlighted by the choice of γ. According to this model, there is a slight increase in MMSE before the accelerated decrease. With regard to the MMSE in practice, this shape may not be realistic. The polynomial model was formulated using the offset parameter ν and the fit in the Fig. 1 shows that this model can describe data where MMSE scores stabilise after a change.

### 2.3 The conditional distribution of the response

In random-effects linear models, the normal distribution is often used for the conditional distribution of the response even in cases where the response is a discrete variable with a limited number of possible values. In a generic notation, this implies $Y|t∼N\left(\eta ,\sigma^{2}\right)$, with η the predictor for the mean, and variance $\sigma^{2}$.

As an alternative, we will discuss two discrete distributions specifically aimed at the situation where the response variable is the (discrete) sum score of a cognitive test with range 0 up to n.

The first distribution is the binomial with the logit link $\pi =l^{*}\left(\eta \right)=\exp\left(\eta \right)/\left(1+\exp\left(\eta \right)\right)$. The distribution is denoted by Y|t∼B(π,n), were π is the success for the n Bernoulli trials. For this well-known distribution, E[Y|t]=nπ and Var[Y|t]=nπ(1−π). The link function l at the start of Section 2 is defined by $l\left(\eta \right)=nl^{*}\left(\eta \right)$.

The second is the beta-binomial distribution which is a combination of two distributions. Assume, firstly, that Y has a binomial distribution with parameters π and n, and, secondly, that π has a beta distribution with parameters $\nu_{1},\nu_{2}>0$. Then the marginal probability distribution function for Y is given by

$$P\left(Y=y|n,\nu_{1},\nu_{2}\right)=\left(\begin{array}{c} n\\ y \end{array}\right)\frac{B\left(\nu_{1}+y,n+\nu_{2}-y\right)}{B\left(\nu_{1},\nu_{2}\right)}\text{,}$$

where $B\left(\nu_{1},\nu_{2}\right)$ is the beta function (Agresti, 2002). Given definitions $\mu =\nu_{1}/\left(\nu_{1}+\nu_{2}\right)$ and $\phi =1/\left(\nu_{1}+\nu_{2}\right)$, the beta-binomial is denoted by Y|t∼BB(μ,n,ϕ) and has E[Y|t]=nμ and Var[Y|t]=nμ(1−μ)[1+(n−1)ϕ/(1+ϕ)]. See also Molenberghs and Verbeke (2005, Section 13.4). The link function l is the same as for the binomial distribution.

Assuming a binomial distribution for the sum score of a cognitive test is in most cases an approximation of the process that leads to the sum score. In the MMSE, for example, the answers to a series of binary questions are strictly speaking not a series of independent Bernoulli trials due to some dependency between the questions. That the trials may not have the same success probabilities does not invalidate the binomial distribution assumption (McCullagh and Nelder, 1989, p. 103).

In a fixed-effects model, the beta-binomial distribution can be used when there is overdispersion with respect to the binomial distribution. In random-effects models the role of the beta-binomial as an alternative is more subtle. If there is an observation-specific random effect in a binomial regression model, then switching to a beta-binomial model does not make sense as the overdispersion is dealt with by the random effects. However, in a model for longitudinal data with individual-specific random effects (some of which are linked to more than one observation), using the beta-binomial distribution may improve analysis.

## 3 Statistical inference

The following discusses maximum likelihood estimation of the change point models, model comparison, the estimation of random effects, and the assessment of residuals.

### 3.1 Semi-parametric maximum likelihood

Longitudinal data are given by $y=\left(y_{1},\ldots ,y_{N}\right)$ and $t=\left(t_{1},\ldots ,t_{N}\right)$, where N is the number of individuals in the sample. For each individual i, we have $y_{i}=\left(y_{i1},\ldots ,y_{in_{i}}\right)$, and observation times measured in years to death $t_{i}=\left(t_{i1},\ldots ,t_{in_{i}}\right)$, where $n_{i}$ is the number of observations for individual i. We assume conditional independence in the sense that $p\left(y|\beta ,\tau \right)=\prod_{i=1}^{N}p\left(y_{i}|\beta_{i},\tau_{i}\right)$, where p(⋅) is a generic notation for a probability density function or a probability mass function, $\tau =\left(\tau_{1},\ldots ,\tau_{N}\right),\beta =\left(\beta_{1},\ldots ,\beta_{N}\right)$ and $\beta_{i}=\left(\beta_{i0},\beta_{i1},\beta_{i2}\right)$ for i=1,…,N. The conditioning on t is ignored in the notation for ease of exposition.

A common choice for the distribution of $\beta_{i}$ in a random-effects model is the multivariate normal. As an alternative, we use non-parametric maximum likelihood (NPML) estimation where the distribution of $\beta_{i}$ is discrete on an unknown finite number K of mass points $z_{k}$, with masses $\pi_{k}$. The likelihood conditional on τ is given by

(5) $$p\left(y|\tau ,\pi ,z,K\right)=\overset{N}{\underset{i=1}{\prod }}\overset{K}{\underset{k=1}{\sum }}\pi_{k}p\left(y_{i}|\tau_{i},z_{k}\right)\text{.}$$

For the individual change points $\tau_{i}$ in τ, we assume a parametric distribution to allow for heterogeneity as well as for pooling of information across individuals. Combining this with (5), the likelihood is given by

(6) $$p_{1}\left(y|\pi ,z,K,\tau_{0},\sigma \right)=\overset{N}{\underset{i=1}{\prod }}\int \overset{K}{\underset{k=1}{\sum }}\pi_{k}p\left(y_{i}|\tau_{i},z_{k}\right)p\left(\tau_{i}|\tau_{0},\sigma \right)d\tau_{i}\text{,}$$

where $p\left(\tau_{i}|\tau_{0},\sigma \right)$ is the distribution for the random change points.

The choice of the distribution for the random change points is of course a model assumption and will depend on the process that is under investigation. In the application, the distribution is specified as a normal distribution with mean $\tau_{0}$ and standard deviation σ, which is truncated at upper bound U equal to zero (time of death) and lower bound L equal to a fixed number of years before death. By varying the specification of the bounds, the sensitivity of the results will be investigated. Choosing a parametric population distribution for the change points, makes it possible to pool information: instead of estimating change points individually, the parameters of their distribution are estimated.

The mass points, the masses, and the parameters for the change point distribution are estimated by maximizing the likelihood (6) conditional on fixed values for K. Transition parameters γ,ϵ or δ can be added to the model as free parameters, but the identification of these parameters along with the other parameters may not always be possible. If data around the change point are sparse, the transition parameters will be hard to identify.

For models with linear predictors, i.e., without change points, there is R-software for NPML. One can use the package npmlreg (Einbeck and Hinde, 2009) for the normal and the binomial model, or the package gamlss (Rigby and Stasinopoulos, 2005) for the normal, the binomial, and the beta-binomial model. These packages use an EM algorithm, which is formulated in detail in Aitkin (1999).

The semi-parametric non-linear random change point model with likelihood (6) was programmed in R, where the trapezoidal rule was applied to approximate the integral and the multi-purpose optimiser optim with the Nelder–Mead algorithm was used to find the maximum likelihood estimate (and the corresponding Hessian). Starting values for the mixture components were derived from the models with the linear predictors fitted in gamlss. Even if the predictors are not truly linear, the estimated masses in the models with the linear predictors will be a good starting point for the estimation of the change point models.

### 3.2 Model comparison

The standard likelihood ratio test for model comparison cannot be applied to the models in this paper. There are two problems. First there is the complication due to using NPML where models with different choices for K are not nested. This is discussed briefly in Aitkin et al. (2009). It is the more general problem of determining the distribution for the likelihood ratio test statistic in mixture models. In the application, we use the Bayesian Information Criterion (BIC) to choose K. The BIC is defined as −2logL+rlog(N), where L is the maximised loglikelihood, r is the number of parameters, and N is the number of individuals, see also Muthén and Asparouhov (2009) who use the BIC in a comparable setting. For longitudinal data, some researchers choose N in the BIC to be equal to the total number of observations. The definition of the BIC is for N equal to the total number of independent observations. Hence both of the above choices are not optimal. See Carlin and Louis (2009) for a discussion of this issue and further references.

The second problem with the likelihood ratio test is with respect to the comparison of a change point model with a model without a change point. A linear model can be described as a degenerated change point model, but this does not produce a framework of nested models. Consider the broken-stick model (1): if the restriction is $\beta_{1}=\beta_{2}$, then τ drops out of the model. If the restriction is τ=0, then $\beta_{2}$ drops out of the model with the additional problem that the hypothesised value of τ is on the boundary of the parameters space.

Model comparison for mixed-effects models is an area of ongoing research. Even for linear mixed-effects model, the often used marginal Akaike information criterion has been shown to be a biased estimator of the Akaike information, see Greven and Kneib (2010).

To the best of our knowledge, there is no theoretically justified way of statistically testing our change point models against alternative models with linear predictors. In the application, we will use residuals plots and rely on large differences between BICs as indicators of better performance.

### 3.3 Fitted values and residuals

Initially, we discuss fitted values and residuals in NPML models before we turn to the semi-parametric change point model. This topic has not yet been fully investigated for NPML models. Aitkin et al. (2009) do not discuss residual diagnostics for NPML, for example. The software gamlss produces plots for randomised quantile residuals (Dunn and Smyth, 1996), but only for mixture distributions that are fitted using estimated marginal mixture probabilities. The following advocates using estimated individual-specific mixture probabilities for residual diagnostics.

Fitted values in an NPML model can be assessed at two levels. Marginally fitted values are computed using the marginal mixture probabilities (the masses $\pi_{1},\ldots ,\pi_{K}$), and within-group fitted values are computed using individual-specific mixture probabilities. The term *within-group* is used in a similar way in parametric random-effects models. In our case, the group consists of the observations within one individual.

A within-group mixture probability is denoted $w_{ik}$ and is the probability that the observations in vector $y_{i}$ come from component k. For individual i, define m^ik=∏j=1nip(yij|z^k), where p(yij|z^k) is the density defined by the NPML model given the estimated mass points $z_{k}$. The estimator of $w_{ik}$ is given by

(7) w^ik=π^km^ik∑l=1Kπ^lm^il,

see Aitkin et al. (2009, Section 9.3). The corresponding within-group fitted values are now defined as y^ij=∑k=1K^w^ikl(η^ijk). The marginally fitted values are obtained by replacing w^ik with π^k.

In the data analysis, we will discuss the (randomised) quantile residuals (Dunn and Smyth, 1996) that are defined using the within-group fitted mixture distribution. Due to the link function in our models assessing directly the difference between observed values and the within-group fitted values is of limited value as there is no obvious distribution for these differences. The quantile residuals, on the other hand, should follow a standard normal distribution if the model is correct. Randomization is used when the distribution for the response is discrete.

The fitted mixture distribution for observation $y_{ij}$ is F^(yij)=∑k=1Kw^ikF(yij|z^k), where F is the chosen cumulative distribution function for the response. The quantile residual is defined as rq,ij=Φ−1(F^(yij)), where Φ is the cumulative distribution function of the standard normal. If F^ is not continuous we follow the approach in Dunn and Smyth (1996) and define aij=limy↑yijF^(y) and bij=F^(yij). The randomised quantile for $y_{ij}$ is then defined by $r_{q,ij}=\Phi^{-1}\left(u_{ij}\right)$, where $u_{ij}$ is a uniform random variable on the interval $\left(a_{ij},b_{ij}\right]$.

In the semi-parametric change point model, the likelihood (6) is marginally defined for the random change points given the marginal NPML mixture probabilities. The estimation of the individual-specific change points can be undertaken by maximum a posteriori (MAP) estimation. The MAP terminology originates from Bayesian inference, where the posterior mode is equal to the maximum likelihood estimate when the prior density is vague and uniform, see, e.g., Rabe-Hesketh and Skrondal (2009) who discuss the estimation of random effects in a generalised linear mixed-effects model.

The posterior of $\tau_{i}$, for i=1,…,N is given by

(8) p(τi|z^,π^,K^,τ^0,σ^,y)∝p(y|z^,π^,K^,τ^0,τi,y)p(τi|τ^0,σ^).

MAP estimation can be performed by maximizing (8) using a multi-purpose optimiser (such as optim in R) conditional on point estimates of model parameters $z,K,\tau_{0},\sigma$, and π.

Conditional on estimated $\tau_{i}$, for all i, within-group mixture probabilities are estimated as explained above for the NPML model. Using these mixture probabilities the (randomised) quantile residuals are defined using the within-group fitted mixture distributions. Plotting the quantile residuals against the fitted values can help to detect outliers or model misfit.

The above definition of randomised quantile residuals ignores possible correlation due to the repeated measurements within individuals. Since the mixed-effects model tries to fit individual growth curves to data within individuals, strong correlation between within-individual residuals is an indication of model misfit. For this reason, it is recommended to assess the residuals within individuals by looking at residual plots per individual. For example, if for many individuals all residuals are positive (or all are negative), then this indicates high within-individual correlation.

## 4 Latent class models

Previous methodology can be extended to a latent class analysis to examine the population for structural differences with regard to the change of cognitive function over time. Random-effects take individual heterogeneity in the data into account and allow for individual trajectories. However, data analysis may improve by explicitly distinguishing latent classes in the data and fitting separate models within these classes.

The following describes a two-level mixture model. The first level is the mixture for a class with change in cognitive function over time (change class) and a class with no change (stable class). The second level consists of a NPML mixture model within each class. Assume that the parameter vectors for the classes are given by $\Delta_{1}$ and $\Delta_{2}$ respectively. Then the likelihood is given by

(9) $$L\left(\theta ,\Delta_{1},\Delta_{2}\right)=\overset{N}{\underset{i=1}{\prod }}\left[\theta p_{1}\left(y_{i}|\Delta_{1}\right)+\left(1-\theta \right)p_{2}\left(y_{i}|\Delta_{2}\right)\right]\text{,}$$

where the mixture proportion θ∈(0,1) is the probability to be in the change class. For the change class, we use the semi-parametric change point model with K components as specified in (6). For the stable class we specify an NPML mixture with $K^{*}$ components. This further specifies (9) as

(10) $$L\left(\theta ,\pi ,z,K,\tau_{0},\sigma ,\pi^{*},z^{*},K^{*}\right)=\overset{N}{\underset{i=1}{\prod }}\left[\theta \int \overset{K}{\underset{k=1}{\sum }}\pi_{k}p\left(y_{i}|\tau_{i},z_{k}\right)p\left(\tau_{i}|\tau_{0},\sigma \right)d\tau_{i}+\left(1-\theta \right)\overset{K^{*}}{\underset{k=1}{\sum }}\pi_{k}^{*}p\left(y_{i}|z_{k}^{*}\right)\right]\text{,}$$

The latent class model allows us to investigate the individual trends in the change class without possible confounding caused by individuals in the stable class.

With respect to the residuals, after the estimation of the model parameters, we first allocate individuals in the sample to either the change class or the stable class by estimating individual class probabilities using MAP. Second we proceed as in Section 3.3 and derive quantile residuals using within-group fitted mixture distributions.

Extensions to more than two latent classes can be defined in a similar way. Although latent classes are easy to formulate in this context, estimation is computationally difficult as each of the classes has its own set of NPML parameters.

## 5 Analysis

The Origins of Variance in the Old–old (OCTO-Twin) study is a population based longitudinal study of Swedish twins in old age (McClearn et al., 1997). Initially, 737 pairs aged 80 years or more were sampled from the Swedish Twin Registry. The pairwise response rate, apart from non-response due to the death of one or both twins in a pair (188 pairs), was 65%, resulting in 351 intact twin pairs aged 80 or older (702 individuals). These individuals were first interviewed between 1991 and 1993 and then at four further interviews conducted at two-year intervals. At each interview the Mini-Mental State examination (MMSE) was used to examine global cognition. Hence the n=30 is the number of trials in the models with the binomial and beta-binomial distribution.

We remove the data for 4 individuals where the MMSE sum score is missing and/or there is no time of observation. In the group of 698 remaining individuals, there are 42 people with no death time - these are the survivors and their data (6%) are removed as well. The data of the remaining N=702−46=656 individuals consist of 2024 records and these will be used in the analysis. The frequencies of the number of interviews per individual are 130,137,116,93, and 180, for number of interviews: 1,2,3,4, and 5, respectively.

As stated in the Introduction, the modelling in the application is tailored to the terminal decline hypothesis which states that individuals in the older population experience a change in rate of decline in cognitive function before death. Of interest is how many years before death this change occurs. To investigate this we will fit change point models on years to death. We will compare the performance of the change point models to the performance of models with linear predictors, and, in addition explore extensions to latent class analysis.

### 5.1 Models with linear predictors

In addition to the non-linear predictors, we also define the linear predictor given by

(11) $$\eta_{L}=h_{L}\left(t,\beta \right)=\beta_{0}+\beta_{1}t+\beta_{2}t^{2}\text{.}$$

Because of the quadratic term, this model is often called a quadratic model, but the predictor is of course linear in the coefficients. This model and its random-effects version can be found in many statistical textbooks.

The OCTO data has sample size N=656. Data are analysed using the linear predictor $\eta_{L}$ in (11). We fitted a series of NPML models for fixed K=4. The motivation for the choice of K=4 is that four components allow for a reasonable amount of individual heterogeneity and at the same time limit the computation burden of maximizing the likelihood. We investigated the normal, the binomial, and the beta-binomial distribution for the MMSE as response, and we tested the restriction $\beta_{2}=0$ in (11).

In the model with the normal distribution, the link function is the identity link l(η)=η. For the binomial and the beta-binomial the logit link was used. The non-parametric maximum likelihood is defined with K=4 components for the random regression coefficients $\beta_{i0}$ and $\beta_{i1}$. Coefficient $\beta_{2}$ is modeled as a fixed-effect. These models are fitted using the software gamlss in R.

For all three distributions, not restricting $\beta_{2}$ yields a better fit in terms of the BIC. For the normal, the binomial, and the beta-binomial distribution, the BIC for the models with unrestricted $\beta_{2}$ are 12 303, 11 905, and 10 541, respectively. See also Table 1.

**Fig. 2:**
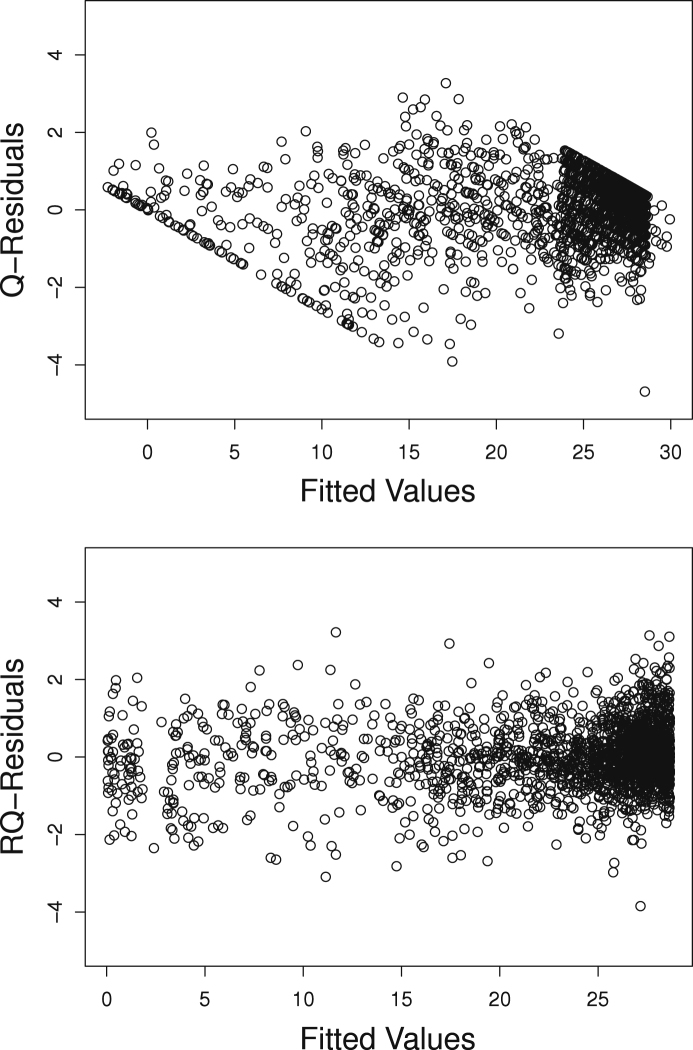
Quantile residuals for the K=4 NPML models with linear predictors including a quadratic term. Model with normal distribution (left) and model with beta-binomial distribution (right).

The top panel of Fig. 2 shows that there is a problem with the normal distribution. The minimum of the fitted values (−2.28) is outside the 0–30 range of the MMSE and the quantile residuals are not independent from the fitted values. The two diagonal bounds in the graph are explained as follows. Given that 0≤y≤30, residual ry^=y−y^ in the original scale has boundaries −y^≤ry^≤30−y^. Switching to quantile residuals re-scales the residuals and the corresponding boundaries as is shown in Fig. 2.

The bottom panel of Fig. 2 depicts the randomised quantile residuals for the beta-binomial model. There is a clear improvement when using the discrete beta-binomial instead of the continuous normal distribution. The quantile residuals in Fig. 2 are derived from within-group fitted values. These fitted values and the corresponding quantile residuals are not directly provided by gamlss, but can be derived using the model fit produced by gamlss. For the additional code, please contact the first author.

### 5.2 Change point models

The truncation of the normal distribution for the random change points as specified in Section 3.1 is chosen for two reasons. First, if there is a change point, it should be timed before death. This means that $\tau_{i}<0$, for all i, since the time scale is years-to-death with time of death equal to zero. We also assume that $-12<\tau_{i}$. This choice is motivated by the length of the follow-up in OCTO (10 years), but also by our interest in change in years before death: going back more than 12 years is of limited use. Hence, the truncation of the normal distribution is defined by lower bound L=−12 and upper bound U=0.

An additional reason to choose a parametric distribution for the change points is that there is an identifiability problem for individuals whose trends show limited or no change over time. If the first slope and the second slope in the predictor for the mean are the same, a change point is not identifiable. In fact, in such a situation, the change point is merely the point where the two parts of the model meet — its location is not important. In that situation we pool information about the change points across the individuals in the data using a parametric distribution.

The summary statistics for the change point models are presented in Table 1. First a range of broken-stick models are assessed with K=4, then smooth shapes are investigated for varying K.

Change point models without latent classes are defined in Section 3.1. The broken-stick binomial model with K=4 has 17 parameters. For random effects $\beta_{i0},\beta_{i1}$, and $\beta_{i2}$ we estimate 3×4 mass points (4 for each random effect). For the mixture proportions we estimate independent masses $\pi_{1},\pi_{2}$, and $\pi_{3}$. For the parametric distribution of the change point we estimate $\tau_{0}$ and σ. The broken stick beta-binomial model with K=4 has 18 parameters since there is an additional scale parameter ϕ.

Table 1 shows that the binomial broken-stick model fits better than the binomial model with the linear predictor. Also the beta-binomial broken-stick model fits better than its linear counterpart. Both with regard to the global deviance and BIC, the beta-binomial broken-stick model is preferred over the binomial model.

Next the model is extended to allow for two latent classes: a stable class and a change class, see Section 4. Interest lies in the proportion of the population that is subject to change of cognitive function, and in the location of the change point if there is a change. From this it follows that the trends in the change class are of primary interest. The latent class model allows us to investigate these trends without possible confounding caused by individuals with no change in cognitive ability.

The two-class models are defined by the likelihood (10), where the model for the stable class is an intercept-only model. The latter is defined by the logit link E[Y]=nexp(α)/(1+exp(α)) and the binomial distribution for the response variable Y. Intercept α is estimated either as a random effect with a discrete distribution estimated by NPML with $K^{*}=2$ components, or as a fixed effect in which case $K^{*}$ is not defined. We start with $K^{*}=2$ to limit the computational burden, but also because the stable class is not of primary interest. In the broken-stick for the change class we use K=4 NPML components as before. Table 1 shows that in this case the beta-binomial model outperforms the binomial model, that including random effects in the model for the stable class leads to a better fit, and that the latent class modelling produces consistently smaller global deviances compared to the one-class modelling. Although the estimation of the mean $\tau_{0}$ of the change point distribution is similar across the two-class models, there is also some variation, which indicates that the estimation of the distribution is sensitive to the different model choices.

To get an idea of how the weighting of the NPML components works out in the mixture defined by π, i.e., the NPML model for the change class, we define the K×K matrix C by the entries

$$c_{lk}=\underset{\left\{i|i\text{ allocated to change class}\right\}}{\sum }w_{ik}I\left(w_{il}=\max\left\{w_{i1},\ldots ,w_{iK}\right\}\right)\text{.}$$

Matrix C is a summary of the distribution of individual mixture weights. Note that $\sum_{k=1}^{K}w_{ik}=1$ and that each vector $w_{i}=\left(w_{i1},\ldots ,w_{iK}\right)$ is a probability distribution. For example, if $N_{c}$ is the number of individuals allocated to the change class, and the diagonal of C is the vector $\left(N_{c}/K,\ldots ,N_{c}/K\right)$, then this would imply a perfect uniform allocation of the $N_{c}$ individuals over the K components.

Matrix C is re-scaled by dividing the rows by the row totals. This defines a matrix C, which can be interpreted as a classification matrix and measures the discriminatory effect of each of the components. For the two-class broken-stick beta-binomial model with K=4 and $K^{*}=2$, there are 367 individuals (56%) who are allocated (by MAP estimation) to the change class, and we obtain

$$C=\left(\begin{array}{cccc} 0.98 & 0.02 & 0 & 0\\ 0.01 & 0.93 & 0.03 & 0.04\\ 0.01 & 0.01 & 0.78 & 0.21\\ 0.02 & 0.06 & 0.15 & 0.77 \end{array}\right)\text{.}$$

So if we would allocate individuals to mixture components according to the maximum of individual component weights, then this classification would be almost perfect for the first component. In comparison, for those individuals allocated to the fourth component, only 77% of the probability mass is assigned to the fourth component.

Given the good performance of the latent-class beta-binomial distribution, we next define smooth change point models within this modelling framework. We start with fixed transition parameters: γ=1 in the Bacon–Watts model, ϵ=1 in the polynomial model, and δ=1/2 in the bent-cable model. Table 1 shows that for these choices, the global deviances of the models are close. For the polynomial model, adding the offset parameter ν is of limited value, ν^=0.098 with estimated standard error 0.139. The model with the offset has a lower global deviance then the model without the offset but the difference is not large. The polynomial model without offset parameter is the same as the bent-cable model and their global deviances are very close. Given that the models are mixtures of mixtures, small differences in the result of the maximization of the likelihood are to be expected.

Results for the Bacon–Watts model, the polynomial model, and the bent-cable model are similar when we compare global deviances. We choose to develop the bent-cable model further. The shape of the Bacon–Watts model may not be realistic for the MMSE: before the decline, there is a an increase according to this model. Even though an increase in MMSE score is possible (for example, after a temporary drop due to illness, or due to a learning effect), we do not think it is correct to assume that such a change always takes place before the change point. In addition to this, the interpretation of the regression coefficients in the Bacon–Watts model is not straightforward as these coefficients cannot be interpreted directly as slope parameters. Of course, the Bacon–Watts model was not developed to describe change of cognitive function and that the model is not optimal in our setting should not be seen as a criticism.

The regression coefficients in the bent-cable model and the polynomial model are directly interpretable as slope parameters. Since the polynomial model with the offset does not lead to a better model, and the polynomial model without the offset is the same as the bent-cable model, pursuing the latter seems the best choice.

Regarding the estimated mean of the change point distribution in Table 1, note that the interpretation across the different models is not the same. For the polynomial model, the change point is the start of the change from the first linear part to the second linear part. For the bent-cable model, the change point is midway the change between the linear parts. This explains the difference between the estimates for these two models. For the last three bent-cable models in Table 1 the estimation of the change point distribution is very similar.

**Fig. 3:**
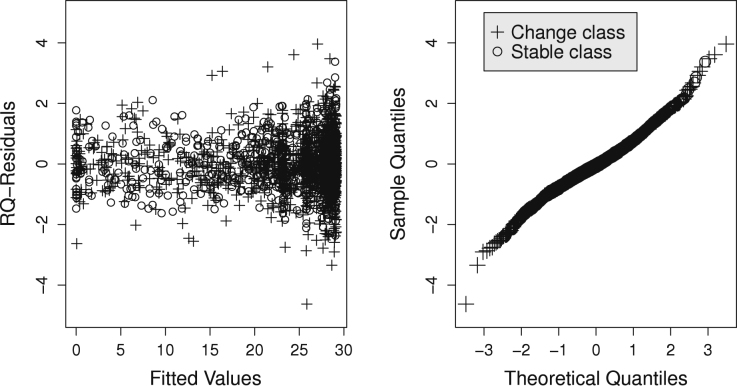
Quantile residuals for the bent-cable model with the beta-binomial distribution, δ=1/2,K=4, and $K^{*}=3$.

There is some benefit in increasing the number of NPML components in the latent class change point models, see Table 1 for the choice $K^{*}=3$ versus K=2 in the broken-stick model and the bent-cable model. However, increasing K=4 to K=5 does not yield a better broken-stick model. We also fitted the bent-cable model for various fixed values of δ. In Table 1, the smallest BIC is 10 339 for the two-class bent-cable model with the beta-binomial distribution, fixed transition parameter δ=3/2,K=4, and $K^{*}=2$. For the same model with δ=1, the BIC equals 10 342. For the two-class bent-cable model with the beta-binomial distribution, δ=1, K=4, and $K^{*}=3$, the BIC is 10 346. For these three models, the BICs are close. However, when we compare quantile residuals, then the third model seems to fit better than the other two producing a Q–Q plot with only a small deviation from the straight line. See Fig. 3 for the randomised quantile residuals diagnostics for the third model. There is some dependence between fitted values and the residuals in the sense that the largest residuals correspond to fitted values at the higher end of the scale. Nevertheless, the residuals do not show a severe deviation from the standard normal. We also looked at within-individual residuals by looking at residuals plots per individual. This was a heuristic test where we looked for instances where all residuals are positive or all are negative. No strong correlation between residuals within individual data was detected.

We choose the model with δ=1/2,K=4, and $K^{*}=3$ as the final model. Parameter estimates are reported in Table 2. In this table, the standard errors are derived from the Hessian provided by the Nelder–Mead optimization, where the delta method was used for those parameters that have a restricted parameter space and were estimated using a transformation. Given the estimated model parameters, we allocated individuals to the two classes using MAP. The number of individuals allocated to the change class is 365, the remaining 291 are allocated to the stable class. Among the 291 individuals in the stable class, there are 63 who are observed only once in the follow-up.

**Fig. 4:**
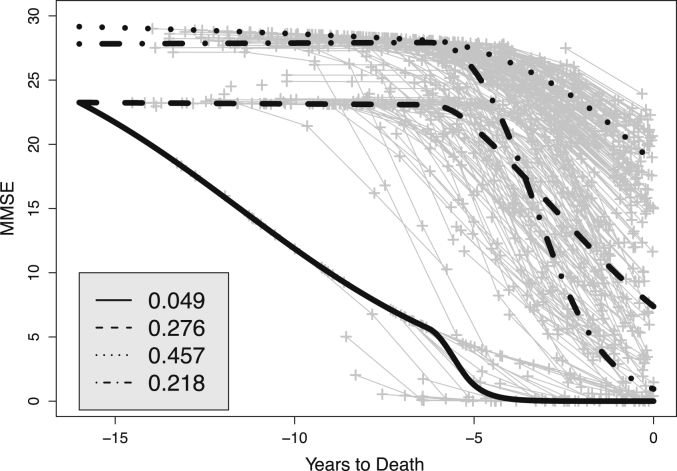
Predicted marginal trajectories (with masses in legend) and individual trajectories for individuals allocated to the change class by the bent-cable model with the beta-binomial distribution, δ=1/2,K=4, and $K^{*}=3$.

Fig. 4 depicts marginal trajectories given the estimated mean of the population distribution of the change point. The classification matrix C for the K=4 component model for the change class is given by

$$C=\left(\begin{array}{cccc} 1 & 0 & 0 & 0\\ 0 & 0.90 & 0.02 & 0.09\\ 0 & 0.01 & 0.98 & 0.01\\ 0.04 & 0.10 & 0.03 & 0.83 \end{array}\right)\text{,}$$

where the entries are rounded. Even though the diagonal entries of C dominate, the individual trajectories in Fig. 4 show considerable heterogeneity. The latter is due to variation in individually estimated change points and to individual-specific mixture probabilities. Even if the estimated intercept and the slopes of the trend of two individuals are the same, the predicted curves for the individuals can still differ due to differences in estimated change points.

**Fig. 5:**
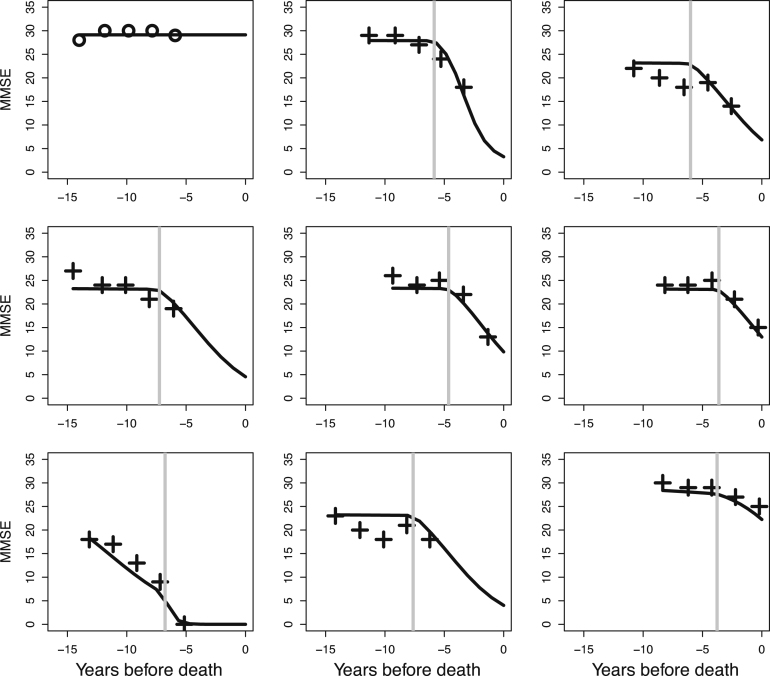
Predicted individual trajectories using the bent-cable model with the beta-binomial distribution, δ=1/2,K=4, and $K^{*}=3$ (+ for change class with vertical line indicating the change point, ∘ for stable class).

Further assessment of the model is possible by plotting estimated curves for individuals in the sample. See Fig. 5 for estimated curves of nine individuals who were randomly sampled among those who had five interviews. Inspecting estimated curves for individuals is worthwhile as it gives insight into the performance of the model on the individual level. This is especially of importance in the current data analysis as it is aimed at the investigation of individual trajectories of cognitive decline.

Fig. 5 shows that the latent-class bent-cable model with the beta-binomial distribution is capable of capturing individual trajectories in the data. The graph in the middle at the bottom in Fig. 5 depicts a situation where the model does not perform well. In general, when observed MMSE scores show an increasing trend at any time point the model will perform poorly, but, according to the assessment of the quantile residuals, the lack of fit of the model in these situations is within reasonable bounds.

Note that although some of the trends may be linear with respect to observed MMSE sums scores, given the bounded scale, we know that trends cannot be linear over the whole time axis. This is nicely shown in Fig. 5, by the graph in the left-hand corner. Because a discrete distribution is used, the extrapolated trend for the last years before death stays within the scale of the MMSE.

As stated before, the choice of the parametric shape of the distribution for the change point is a model assumption. To investigate the sensitivity of the results to the specification of the lower bound L of the truncated normal, we re-fitted the model for L=−10,−15, and −30. The effect of the alternative specifications of the lower bound is limited when compared with the results for L=−12. For example, for L=−10, point estimates of the mean and the standard deviation (μ,σ) of the change point distribution are (−5.931,2.269), for L=−15, we get (−5.853,2.495), and for L=−30 we get (−6.163,2.295).

The analysis was inspired by the terminal decline hypothesis. Of interest was whether it is true that there is a change in rate of decline in cognitive function before death, and if so, how many years before death this change takes place. Our modelling shows that change point models describe the OCTO-twin data better then models with linear predictors. Even when no latent classes are distinguished, the change point models outperform the models with the linear predictors, see Table 1. Adding the latent class modelling allows for further improvement of model fit.

According to the data analysis, not all individuals experience a change, but for those who do (with an estimated prevalence of 65% and estimated standard error of 3%) the mean number of years of this change before death is estimated at 5.8 years (with estimated standard error 0.3). There is some heterogeneity among these individuals as 5.8 years is the mean of an assumed normal distribution (truncated at 12 and 0 years before death) with a standard deviation estimated at 2.4 years.

Note that the estimated change point distribution is hard to compare with results published in the literature. First, the OCTO data are subject to left truncation (only those who survived up to 80 years old were eligible for the study). It may be that change point behaviour is very different for those who die before they are 80 years old, in which case the result of the current analysis should not be extrapolated to those who die before they are 80. Secondly, the choice of years-to-death as time scale further hampers comparison with studies that use age as a time scale. Likewise, one should be careful when comparing our results with results for specific sub-populations, such as people with dementia, see, e.g., Hall et al. (2003) and Bartolucci et al. (2009).

Nevertheless, given that mean age of death in OCTO for those who are allocated to the change class is 90.1 years old, and with the mean of the change point distribution estimated at 5.8, we conclude that, if there is a change, then this change takes place around 84.3 years old on average. (Mean age of death for those allocated to the stable class is 89.6.)

## 6 Conclusion

This paper introduces methodology for change point models for cognitive tests. Specific modelling choices are the beta-binomial distribution for the response variable, and a parametric distribution for the random change point combined with non-parametric distribution for random intercept and slope parameters. Estimation is via maximum likelihood. Model comparison is hampered by the lack of formal tests but can be undertaken heuristically by comparing BICs. Model fit is assessed by quantile residuals diagnostics derived from within-group fitted distributions. To acknowledge that there may be different groups in the sample with respect to cognitive change, an extension to a latent-class model is formulated. In the application, longitudinal data are used from a study where death times are available for 94% of the participants. The data from survivors are ignored in the analysis and the time scale for the models is years to death. Two latent classes are distinguished: one in which individuals experience a change over time in cognitive change, and one in which the cognitive function is stable.

Ignoring data from survivors in the analysis is of course an important issue. When the group of survivors is relatively small, as in our application, the effect of ignoring this group will be negligible. Note that in the application many survivors will be close to death. In other applications where the group of survivors is relatively large, one might consider a joint model where the growth model is combined with a survival model, see Verbeke and Davidian (2009) for an introduction to joint modelling and references.

The definition of the classes was made a priori and was driven by our research question: if there is change, can we say something about individual change points? Hence, beforehand, we decided to distinguish a class with change and one without, and we fitted the trend in the stable class with an intercept-only predictor. Alternatively, we could have looked for the best latent class model for the data, in which case we would have had to estimate the optimal number of classes, and, for each class the optimal class-specific predictor. The presented methodology can be used to go down this route. However, without a sharp subject-matter distinction of the classes, interpretation of results will be hard and may be also difficult to defend. Also, testing for the optimal number of classes is difficult, see Section 2.3.

A model where the distribution of the response is assumed to be normal is problematic for the longitudinal MMSE scores. The main problem is not that the MMSE scale is discrete, but that there is a ceiling effect in the sense that many observed scores are close to the upper bound of the scale. Transforming the MMSE scores to another scale does not solve this problem. We suggest to deal with the ceiling effect by assuming a discrete distribution for the MMSE response.

Using the beta-binomial distribution appreciates the discrete nature of the MMSE and is an improvement compared to using the normal distribution. However, given that there is some dependency between the MMSE questions, the assumption of independent Bernoulli trials is violated. More work is needed to investigate possible impact of this violation. A solution would be to replace the observed sum score by a latent variable which is linked to question-specific scores via an item response theory model, see Fox (2010) who discusses a linear mixed-effects model for longitudinal MMSE data, or Klein Entink et al. (2011). A disadvantage of working with the latent variable is the lack of a straightforward interpretation of the parameters for the regression model.

Related to the idea of using a latent variable is to use a censored regression model for a bounded score with a ceiling effect, see, for example, Hutton and Stanghellini (2011). The censoring in this context implies that the scale for the test is extended beyond the original scale and hence also beyond the range of the data, which we consider to be very problematic with respect to the interpretation of a fitted model.

In the data analysis, heterogeneity between individuals is taken into account by using individual-specific random effects. The modelling is tailored to the terminal decline hypothesis and effects of background variables such as gender and age are outside the scope of the present work. However, these variables can be added as covariates to the regression equations and the optimization of the likelihood can proceed along the lines set out in this paper. Because a multi-purpose optimiser is used, increased model complexity may slow down estimation considerably. A further step would be to formulate a regression model for the mean of the distribution for the change points, but there may not be enough information in the data to identify such a model.

The semi-parametric change point model was programmed in R and the multi-purpose optimiser optim was used to find the maximum of the likelihood. Although it is relatively easy to define extended models, for example by adding covariates or by allowing for more than two latent classes, computing time will increase rapidly. It is advisable to run preliminary analyses with a limited number of NMPL-mixture components and a reduced number of grid points for the trapeziodal rule. Providing starting values is important, but it is not easy to come up with good values from scratch. However, starting values for the mass points in the NMPL can be derived from fixed-effects models fitted to data from one individual. In addition, starting values for the masses can be obtained from fitting models with linear predictors in gamlss.

The parameters of the broken-stick model and the bent-cable model have a clear interpretation. For the Bacon–Watts model the interpretation is more complicated and is one of the reasons why this model was not pursued in the application. A disadvantage of the presented change point models is that there are transition parameters which may or may not be identifiable from the data. Whether this is a problem will vary with the aim of the analysis. In our application, we were not able to estimate the transition parameters from the data. Instead, models were compared for a range of fixed values.

## Figures and Tables

**Table 1:** Models for the OCTO data. The number of NPML components is K. In the two-class models, $K^{*}$ is number of NPML components for the stable class. The number of parameters is nP, and GD is the global deviance. Estimated mean $\tau_{0}$ for the change point distribution.

	K	$K^{*}$	nP	GD	BIC	$\tau_{0}$
*Models with linear predictors*						
Normal	4	–	13	12 218	12 303	–
Binomial	4	–	12	11 827	11 905	–
Beta-binomial	4	–	13	10 456	10 541	–
*Broken-stick change point models*						
Binomial	4	–	17	10 509	10 620	−3.72
Beta-binomial	4	–	18	10 370	10 487	−4.29
*Two-class broken-stick change point models*						
Binomial (α fixed effect)	4	–	19	10 472	10 596	−5.18
Binomial	4	2	21	10 409	10 546	−5.52
Beta-binomial (α fixed effect)	4	–	20	10 246	10 376	−5.72
Beta-binomial	4	2	22	10 226	10 369	−5.07
Beta-binomial	4	3	24	10 193	10 349	−5.61
Beta-binomial	5	2	26	10 222	10 391	−5.26
*Two-class smooth change point beta-binomial models*						
Bacon–Watts (γ=1)	4	2	22	10 221	10 364	−4.68
Polynomial (ϵ=1, w/o offset)	4	2	22	10 226	10 369	−5.08
Polynomial (ϵ=1, w/ offset)	4	2	23	10 214	10 364	−5.17
Bent-cable (δ=1/2)	4	2	22	10 225	10 368	−5.11
Bent-cable (δ=1/2)	4	3	24	10 190	10 346	−5.76
Bent-cable (δ=1)	4	2	22	10 199	10 342	−5.60
Bent-cable (δ=3/2)	4	2	22	10 196	10 339	−5.65

**Table 2:** Parameters for the bent-cable model with the beta-binomial distribution, δ=1/2,K=4, and $K^{*}=3$. Standard errors in parentheses.

Latent-class mixture proportion				θ	0.654	(0.030)		
Change point model for change class								
	*Mass points*							
$\beta_{0}$	−3.194	(0.578)	1.183	(0.232)	2.134	(0.382)	2.634	(0.418)
$\beta_{1}$	−0.277	(0.057)	−0.003	(0.025)	−0.089	(0.048)	0.006	(0.047)
$\beta_{2}$	−1.732	(1.079)	−0.400	(0.040)	−0.286	(0.064)	−1.051	(0.111)
	*Masses*							
	0.049	(0.045)	0.276	(0.040)	0.457	(0.048)	0.218	(0.048)
*Change point distribution*				*Beta-binomial distribution*				
μ	−5.762	(0.334)		ϕ	0.044	(0.005)		
σ	2.392	(0.207)						
Linear model for stable class								
	*Mass points*							
α	1.806	(0.081)	2.663	(0.196)	3.539	(0.149)		
	*Masses*							
	0.235	(0.056)	0.290	(0.114)	0.475	(0.113)		

## References

[br000005] Agresti A. (2002). Categorical Data Analysis.

[br000010] Aitkin M. (1999). A general maximum likelihood analysis of variance components in generalized linear models. Biometrics.

[br000015] Aitkin M., Darnell R.E., Francis B.J., Hinde J.P. (2009). Statistical Modelling in R.

[br000020] Bacon D.W., Watts D.G. (1971). Estimating the transition between two intersecting straight lines. Biometrika.

[br000025] Bartolucci A., Bae A., Singh K., Griffith H.R. (2009). An examination of Bayesian statistical approaches to modeling change in cognitive decline in an Alzheimer’s disease population. Mathematics and Computers in Simulation.

[br000030] Bauwens L., Rombouts J.V.K. (2012). On marginal likelihood computation in change-point models. Computational Statistics & Data Analysis.

[br000035] Carlin B.P., Louis T.A. (2009). Bayesian Methods for Data Analysis.

[br000040] Chiu G., Lockhart R., Routledge R. (2006). Bent-cable regression theory and applications. Journal of the American Statistical Association.

[br000045] Cohen P. (2008). Applied Data Analytic Techniques for Turning Points Research.

[br000050] Dominicus A., Ripatti S., Pedersen N.L., Palmgren J. (2008). A random change point model for assessing variability in repeated measures of cognitive function. Statistics in Medicine.

[br000055] Dunn P.K., Smyth G.K. (1996). Randomised quantile residuals. Journal of Computational and Graphical Statistics.

[br000060] Einbeck, J., Hinde, J., 2009. Nonparametric maximum likelihood estimation for random effect models in R. Vignette to R package npmlreg version 0.44.

[br000065] Folstein M.F., Folstein S.E., McHugh P.R. (1975). Mini-mental state: a practical method for grading the state of patients for the clinician. Journal of Psychiatric Research.

[br000070] Fox J.-P. (2010). Bayesian Item Response Modeling.

[br000075] Greven S., Kneib T. (2010). On the behaviour of marginal and conditional AIC in linear mixed models. Biometrika.

[br000080] Hall C.B., Ying J., Kuo L., Lipton R.B. (2003). Bayesian and profile likelihood change point methods for modeling cognitive function over time. Computational Statistics & Data Analysis.

[br000085] Hutton J.L., Stanghellini E. (2011). Modelling bounded health scores with censored skew-normal distributions. Statistics in Medicine.

[br000090] Kiuchi A., Hartigan J.A., Holford T.R. (1995). Change points in the series of T4 counts prior to AIDS. Biometrics.

[br000095] Klein Entink R.H., Fox J.-P., Van den Hout A. (2011). A mixture model for the joint analysis of latent developmental trajectories and survival. Statistics in Medicine.

[br000100] Laukka E.J., MacDonald S.W.S., Bäckman L. (2006). Contrasting cognitive trajectories of impending death and preclinical dementia in the very old. Neurology.

[br000105] McArdle J.J., Wang L., Cohen P. (2008). Modeling age-based turnings points in longitudinal life-span growth curves of cognition. Applied Data Analytic Techniques for Turning Points Research.

[br000110] McClearn G.E., Johansson B., Berg S., Pedersen N.L., Ahern F., Petrill S.A., Plomin R. (1997). Substantial genetic influence on cognitive abilities in twins 80 or more years old. Science.

[br000115] McCullagh P., Nelder J.A. (1989). Generalized Linear Models.

[br000120] Molenberghs G., Verbeke G. (2005). Models for Discrete Longitudinal Data.

[br000125] Muggeo M.R. (2008). Modeling temperature effects on mortality: multiple segmented relationships with common break points. Biostatistics.

[br000130] Muniz-Terrera G., Van den Hout A., Matthews F.E. (2011). Random change point models: investigating cognitive decline in the presence of missing data. Journal of Applied Statistics.

[br000135] Muthén B., Asparouhov T., Fitzmaurice G., Davidian M., Verbeke G., Molenberghs G. (2009). Growth mixture modeling: analysis with non-Gaussian random effects. Longitudinal Data Analysis.

[br000140] Rabe-Hesketh S., Skrondal A., Fitzmaurice G., Davidian M., Verbeke G., Molenberghs G. (2009). Generalized linear mixed-effects models. Longitudinal Data Analysis.

[br000145] Riegel K.F., Riegel R.M. (1972). Development, drop, and death. Developmental Psychology.

[br000150] Rigby R.A, Stasinopoulos D.M. (2005). Generalized additive models for location, scale and shape (with discussion). Journal of the Royal Statistical Society: Series C (Applied Statistics).

[br000155] Rudoy D., Yuen S.G., Howe R.D., Wolfe P.J. (2010). Bayesian change-point analysis for atomic force microscopy and soft material indentation. Journal of the Royal Statistical Society: Series C (Applied Statistics).

[br000160] Stasinopoulos D.M., Rigby R.A. (1992). Detecting break points in generalised linear models. Computational Statistics & Data Analysis.

[br000165] Tishler A., Zang I. (1981). A new maximum likelihood algorithm for piecewise regression. Journal of the American Statistical Association.

[br000170] Van den Hout A., Muniz-Terrera G., Matthews F.E. (2011). Smooth random change point models. Statistics in Medicine.

[br000175] Verbeke G., Davidian M., Fitzmaurice G., Davidian M., Verbeke G., Molenberghs G. (2009). Joint models for longitudinal data: introduction and overview. Longitudinal Data Analysis.

